# Hospital and Institutional News

**Published:** 1919-11-08

**Authors:** 


### HOSPITAL AND INSTITUTIONAL NEWS.

### A WORD ON THE HOSPITAL CRISIS.

In the present time of crisis for our hospitals it must always be borne in mind that "Scrap the lot" is a phrase which applies in these days to very many institutions and businesses, as well as to Government Departments. All the well managed ones will endure, because the British people have at heart the determination to uphold and continue the voluntary hospital system in this country. Continued pessimism and slackened effort in connection with a given institution must lead it, or rather those responsible for such a policy, rapidly in the direction of the scrap-heap. Indeed the claims and demands of the suffering and the sick, with the present financial burden which the nation has to meet, entail on habitual supporters everywhere the duty of "scrap" and "sack" whenever and wherever the necessity arises to secure the efficiency without which the best of hospitals may approach nearer and nearer to a state of practical financial bankruptcy. Let us save the hospitals as we readily can, if we combine with a will and put our backs into the glorious work of rescue. Wake up, pessimists, or make way for better men! The crisis may prove severe, but victory lies within the power of every worthy man and woman who may hold responsible office in connection with the finances and needs of each well-administered hospital worthy of the name.

### THE GENERAL MEDICAL COUNCIL ELECTION.

During the next few days there will be sent out to each member of the medical profession duly registered in these islands a voting paper for the election of direct representatives on the General Medical Council, the first such election since 1911. There are four vacancies for England and Wales, and already a number of candidates have issued election addresses. As in 1911, the British Medical Association is putting forward four candidates officially recommended by that body, and selected months ago by a vote of the Association's Representative Meeting. As eight years ago, these four will probably secure election, although their election address is delightfully vague in its programme and commits the four to absolutely nothing whatever. An impressive list of local and central officers of the British Medical Association who have promised to support these candidates, accompanies this election address: it contains a sprinkling of well-known names, but can hardly be described as distinguished. Then there are four candidates selected by the Medico-Political Union, who are somewhat vehement, and doubtless very earnest. Whether any more candidates are in the field is so far uncertain: but no other election addresses have been distributed so far, so that a straight fight between the two associations appears probable.

### G. Q. ROBERTS, C.B.E., M.A.OXON.

Under a misapprehension it was erroneously stated in last week's issue of The Hospital that continuous nursing had unexpectedly fallen to Mrs. Roberts' lot. Mrs. G. Quinlan Roberts, we learn, has not been nursing her husband during his recent illness. Mr. Roberts has been skilfully and carefully nursed with unfailing attention in Arthur Small Ward by the sister and two special nurses of the St. Thomas's Hospital staff. Their continuous care since his severe operation has, in God's good providence, ministered to his most satisfactory progress. Mrs. Quinlan Roberts desires to express her thanks to all those who have made enquiry for her husband. It would be impossible to answer separately all the messages of sympathy and condolence in her recent anxieties. It is hoped that Mr. Roberts, who is progressing rapidly, will be able to leave the hospital in the course of the week. This announcement will give great pleasure to many, for a large number of hospital workers and others will welcome this evidence of his improving convalescence, and desire him the best of weather and everything that can minister most certainly to his complete recovery.

### MEDICAL DEFENCE IN SCOTLAND.

The two societies which are established in England for medical and dental defence are well-known and appreciated; each of them has a large and increasing membership. It is, however, not so well known that (though the English societies accept Scottish and Irish members), Scotland possesses a union of its own, known as the Medical and Dental Defence Union of Scotland. Inasmuch as Scotland has a separate, and in many respects a different, code of law from that of England, there is a good deal to be said for this subdivision, though in general terms the multiplication of such associations is to be deprecated on the grounds of administrative expense and lessened financial stability. The seventeenth annual report and statement of accounts has just been issued, from which several points emerge that are worthy of mention. The membership has reached 1408, and is increasing. The last year's working resulted in a small balance of income over expenditure. The reserve fund is in a healthy state, considering the size of the society. Unfortunately, pages 5 and 8 of the report are printed twice over, to the exclusion of pages 6 and 7, a mishap which argues lack of vigilance on somebody's part. Enough remains to show that the union is doing its utmost to compete with the English societies, because it is offering to accept transfers of membership from these without entrance fee, and offers other temptations. The wisdom of this competitive action is doubtful: one of the chief advantages of a separate society for Scotland is familiarity with Scottish law, an advantage which it at once loses by starting to cater for medical men practising in England.

### DEATHS FROM INFLUENZA.

We have read in recent issues of more than one medical journal paragraphs making light of the recurrence of influenzal infection in the country, and roundly criticising the lay press for its "harping" on the old warnings and precautionary measures. In these pages, to the still present menace of influenza, attention has been repeatedly drawn, and it seems a great pity that, because no great death-dealing epidemic has appeared this autumn, the impression should be circulated that need for all precautions is past. Figures taken from the greater towns of England and Wales show that since August there has been a steady increase in the weekly number of deaths from influenza; in that time the death-rate has, in fact, been multiplied four times. The deaths from all forms of pneumonia in London alone during the week ending October 25, were 98. It is far from our intention to produce an unnecessary scare, but we nevertheless heartily commend the action of those lay papers which have seen fit to attempt a simple medical explanation for the public of the present danger in catarrhs, and we think it most surprising that professional journals should attempt to ridicule the importance of such efforts.

### PRACTITIONERS AND TROPICAL DISEASES.

ALTHOUGH we are not in receipt of details of the scheme, we understand that, under the Ministry of Pensions organisation, specialists in tropical disease are about to visit various areas in the country, with a view to affording assistance to the local practitioners in dealing with the many post-war cases of infections relatively new to the English countryside. Although trench fever does not, perhaps, fall strictly under this category, yet there is urgent need to better understanding of the late manifestations of this condition, and it is sincerely to be hoped that opportunity will be taken to produce a more widespread appreciation of its symptoms. It is now some months since malaria, dysentery, trench fever, etc., became notifiable, but, as was widely demonstrated at the time, it is quite impossible to attach value to such notification when the doctors concerned are in many cases unable to make anything approaching a certain diagnosis. The policy of education is the only one which can bring protection to the country and justice to the unfortunate patient, and the new scheme has our unqualified approval.

### THE SPAN OF HUMAN LIFE.

IN a connection quite other than that of glandular activity, this subject is again brought up by a recent address of Dr. William Mayo, the President of the American College of Surgeons. He tells us that, since the end of the American Civil War, fifteen years have been added to the average length of human life, and states that it is certain that, after the next twenty years, another ten will be added to the span. The last few years have proved how invaluable to a country are its older men—this during what has been described as a young man's war; but that the natural cure for industrial unrest lies in this life-lengthening process does not to us appear, at first sight, obvious. It is true that in higher vocational spheres plentiful matured skill and experience will do much to improve a nation, and among the masses the older men are probably less easily impressionable, and their own responsibilities hold them back from the influence of the numerous agitators with which every land is nowadays so unfortunately afflicted. Dr. Mayo's words open up a most interesting line of speculation as to the nations of the future.

### "A BAD PRECEDENT."

DR. KEITH S. RODEN has been appointed Resident Medical Officer at Worcester General Infirmary, at a salary of £200 a year, with board, lodging, and washing, in succession to Dr. Allport. The post had not been advertised, because, Mr. G. Shrewsbury Smith declared, Dr. Roden had applied before an advertisement had been published. Dr. Moore Ede, the Dean of Worcester, said that a bad precedent had been created. It was then stated that an application to St. Bartholomew's Hospital had brought the reply that the terms on which the post was offered made it "utterly hopeless" to expect applications, because the Army was now asking for young practitioners to whom a salary of £600 to £700 a year was open. This explanation, it is reported, was considered "satisfactory." Before this view can be endorsed, however, some further consideration is desirable. Dr. Allport, it was decided at the previous executive meeting, should be given three months' notice. He, however, wished to resign on December 31; in consequence it was decided to withdraw the notice. Then came Dr. Roden's application for the post. How did he, in Droitwich, know of the vacancy at Worcester at the moment when it occurred; for his application, from the point of view of the committee and of himself, was received with operatic opportuneness? We suggest no indiscretion on anybody's part. But our memory of how a certain appointment at a different institution eventually became the subject of a public inquiry, suggests that, in the interests of the doctors and of the infirmary, this apparently trivial coincidence should be explained. Meantime what is the view of the honorary medical staff upon the salary and the opinion implied thereon by the authorities of St. Bartholomew's Hospital?

### THE SOLDIER'S TONGUE.

IF the average private could see in print the language which he is accustomed to utter, he would be almost as much surprised as are the old ladies who chance occasionally to overhear his oaths. Print would bring his idiom home to him. But the language which he uses to his fellows is rarely heard in hospital wards. Lately, in fact, Colonel Trimble, who was head of a hospital at Etaples, referred to this curious army *argot*, but added that, in all his experience of 40,000 military patients, he had never heard a man, or even of a man, who used a coarse word in the wards. If this is typical it shows how easily man can divide his life, his actions, and his words into watertight compartments, and how completely he can keep one side of his life free from the overt influence of another. It is a dangerous feat. The honest man, the wise man, is not one who, for instance, will pay tribute to charity on Sunday and cheat his customers across the counter during the week. The hospital officer or nurse who is a different person off duty from that which he or she is in the office or the wards is unworthy of a great profession. We may hope that life in a hospital did something for soldiers in civilising many who, but for the war, might never have come within the range of its influence.

### MAKING MOSS WHILE THE SUN SHINES.

The autumn rains are due, but, at the moment of writing, we are still enjoying Michaelmas summer. Hospitals, which touch life at every point, are concerned in the recent fine weather because it provides the last opportunity to collect sphagnum moss before the autumnal deluge. In Hampshire collectors have been invited to make moss while the sun shines, other householders have been asked to present muslin, and sacks have been promised from the Royal Hampshire County Hospital for those collectors of sphagnum moss who have secured enough to fill them. However the supplies of dressings may improve, we hope that the policy of collecting moss and inviting gifts of kindred materials will not be allowed to lapse. For many enjoy collecting moss who dislike extremely collecting money, and the business of a voluntary hospital is to share all round the fun and good-fellowship of which it is the centre.

### THE COST IN CASUALTIES OF THE STRIKE.

The strike has affected hospitals chiefly perhaps in bringing unexpected accidents to their doors, particularly in the case of those situated in the country districts. There the internal-combustion engine makes the roads hum and picks up all sorts of stray travellers, not to mention their parcels. The additional omnibuses cannot properly supply the demands. With more good humour than safety, standing room is offered to everyone. The consequence is that some people fall off their precarious perches, and farmhouses receive patients with broken limbs or minor injuries, and these have to be carried on carts to the nearest hospital. On the whole in this sense the strike has provoked more good-humour than injuries, but the latter have been frequent, and have revived the more serious side of the old humours of the road.

### THE ROYAL LIVERPOOL CHILDREN'S HOSPITAL.

The already announced amalgamation between the Liverpool Infirmary for Children, and the Royal Liverpool County Hospital for Children has led to a rechristening of the new joint institution under the title which heads this note. Mr. E. J. Deane, the Secretary of the County Hospital, in seeking to enlist support, reminded the public that the completion of the County Hospital at Heswall was interrupted by the war. A sum of £70,000 is needed for this and to provide additional room at Myrtle Street. The end in view, which is a worthy one, is to make the two hospitals thus fused the best centre of study for infant diseases in the North.

### SOUTHAMPTON'S CHILDREN'S HOSPITAL.

It has been decided to change the name of the Children's Hospital and Dispensary at Shirley, Hants, to the "Southampton's Children's Hospital and Dispensary for Women." Help and patients come from beyond Shirley, and the rechristening of the hospital fulfils an ancient hope of Dr. Saunders, who has favoured it consistently.

### THE PEACE PARTY AT DONCASTER.

Now that an accommodation has been reached between the committee and the medical staff of Doncaster Royal Infirmary, it is wiser not to detail the causes of the lately ended friction. This, as usual, reposed on innuendo, and to track its course is as sorry a business as to follow the slime of a slug from the sink-trays to the kitchen. The argument was reached after a round-table conference had been held, and the final amity was distinguished by a decision to re-elect the whole seventeen members of the committee. Thus the party of peace carried the day and, since the committee was divided against itself as well as opposed to the medical staff, Mr. W. Clark's proposal of a re-election *en bloc* and its subsequent adoption was a very happy termination of the difficulty.

### NEW ALBANY HOSPITAL, GRAHAMSTOWN, S.A.

At the September meeting of the Albany Hospital Board, Grahamstown, South Africa, when Mr. H. R. Wood presided, a letter was read from the Cape Provincial Secretary enclosing a copy of a memorandum from the Public Works Department giving an estimate of the cost of the scheme for the new Albany Hospital, and providing for certain alterations and omissions. This brought the estimated cost of the new hospital down to £57,000, and His Honour the Administrator has authorised the invitation of tenders on the understanding that the board will collect the further funds required after allowance is made for a further loan of £10,000, which amount is the limit of assistance to be derived from the Cape Provincial Administration. Mr. Piggott pointed out that the board had practically £40,000, and the Administrator was willing to give the board a further £10,000 provided the board guaranteed another £10,000 also. Thus they had £60,000, although the plans were reduced to £57,000.

### A "LEGAL" QUALIFICATION FOR DISPENSERS

Mr. A. H. Jenkins, Chairman of the Public Pharmacists Association and a member of the Council of the Pharmaceutical Society, contributes a very thoughtful article on the conditions governing dispensing of medicines in Great Britain to the *Pharmaceutical Journal*. Except for "National Insurance Dispensing" no qualification is *legally* requisite for a person who dispenses medicine, even containing poison, and unless "sold" in an open shop no law is contravened. Briefly, Mr. Jenkins's scheme is to have a special qualification for "Dispenser," obtained after a curriculum on the lines of that required for a "Pharmacist," with the exception that training may be obtained in a hospital dispensary instead of a retail chemist's. On passing a further examination the "Dispenser" could (after experience in a retail shop) have the right of opening a chemist's business. The matter is one which cannot be dealt with except by legislation, but, now that the Ministry of Health scheme is under way the time seems opportune to remedy the defect concerning such an important matter. It is to be noted that in Ireland the law requires compounding of medicines to be done by properly qualified pharmaceutical chemists.

### RAISING THE STANDARD OF BERMONDSEY INFIRMARY.

Under the superintendence of Dr. F. C. Harkness, who six months ago was appointed superintendent of Bermondsey Infirmary, efforts are being made to raise the standard of the infirmary to the level of a general hospital. The committee are working in active sympathy with Dr. Harkness, and are continuing the splendid work done at the infirmary during the many years that the late Dr. E. S. Bell was superintendent. Dr. A. Salter, whilst a member of the board, did much to bring the infirmary up-to-date, and, as his place has been taken by his wife, Mrs. A. Salter, there is little doubt that Dr. Harkness will be able to achieve some notable alterations which will alike benefit the staff and the patients. As a preliminary move application is to be made to the Ministry of Health for permission to alter the name of the infirmary to that of "The Bermondsey and Rotherhithe Hospital."

### ONE WAY OF GETTING A SECRETARY.

When the history of the auxiliary war hospital movement finds a chronicler, he or she will have to devote an early chapter to the Hospital for Officers started by Miss Keyser ("Sister Agnes") during the South African War, and kept in being right down to the present day. By certain announcements in the "Agony column" of the *Times*, the public learnt last week that this, perhaps the best-known of all the London auxiliary hospitals run by private individuals, has moved permanently to Grosvenor Crescent from the home it has occupied so long in Grosvenor Gardens. The next paragraph inserted asked (a shade pathetically) whether anyone could recommend a gentleman for the post of paid secretary to this institution; and a few days later yet a third paragraph announced that "Sister Agnes has engaged a secretary," and that the letters she received are too numerous to answer personally. From these statements it may be inferred that Sister Agnes intends to keep her hospital in being and not to demobilise it, which is the same course that she pursued after the South African War. The reputation of this hospital has always stood very high in all respects; in discipline, especially, a standard is maintained possibly loftier than that of any other similar organisation. The name of the gentleman selected as secretary was not published.

### THE COTTAGE HOSPITAL MOVEMENT.

It is encouraging to report the progress being made with new cottage hospitals. The foundation stone of the North Walsham and District War Memorial Cottage Hospital, the site of which was a gift from Mrs. John Wilkinson, was laid last month. The hospital will contain two wards with three beds and one cot apiece, and two private wards with a single bed in each of them. There will also be three bedrooms. The land is about an acre in extent, and includes a sandpit, which is to be converted into a rock garden and to be provided with a shelter. The cost of building is expected to be £4,000 and a similar sum is required for its partial endowment. At the ceremony, Sir H. A. Ballance recalled that the first cottage hospital was built in Surrey exactly sixty years ago, under the name of a village hospital. More cottage hospitals were needed in Norfolk, and the movement has evoked the admiration of Americans. In Shropshire, the parish of Chirk is endeavouring to establish a cottage hospital for the district. Mr. A. Wood is secretary of the committee. It is also satisfactory to learn that cottage hospitals are benefiting by the distribution of the Red Cross surplus. Thus Petersfield Cottage Hospital has been allotted £2,000 and will receive a further £800 from the Hampshire Demobilisation Committee. Cottage hospitals work quietly and well, and it is gratifying to note their progress and its recognition.

### BOOMING WELFARE WORK.

In these days when Government Departments have Press agents, local authorities are finding it advisable to boom various branches of municipal enterprise. They already advertise venereal disease treatment facilities and now the Durham County Council is taking steps to bring to the knowledge of the public the various schemes in respect of maternity, child-welfare, tuberculosis, midwifery service, and health visiting. The County Medical Officer of Health has asked the members of the County Council to convene public meetings in the constituencies so that they, with members of the public health staff, can tell the audiences of the health possibilities, etc., which are at their service.

### THIS WEEK'S DRUG MARKET.

Generally speaking prices are well maintained and there are no signs of any impending widespread break in values; on the contrary, the tendency of prices is in many cases in an upward direction, being due in most instances to inadequacy of supplies, but in a few cases partly to manipulation. Menthol still moves upwards, and the high value of camphor is firmly maintained. Any ergot of rye available fetches remarkably high figures, and there seems to be no immediate prospect of the scarcity being relieved. Clove oil moves up in value with the advancing price of cloves. The value of Turkey opium has an upward tendency. The price of lemon oil has advanced substantially. Quinine is still under control, and the controlling authority has made it clear that sales above the controlled price are illegal. In view of recent arrivals of rhubarb it is possible that there may be some falling off in price, which is remarkably high, sulphonal is cheaper. Antifebrin, resorcin and hexamine have an upward price tendency.

